# Early Parenting Program as Intervention Strategy for Emotional Distress in First-Time Mothers: A Propensity Score Analysis

**DOI:** 10.1007/s10995-012-1088-6

**Published:** 2012-08-05

**Authors:** Miwako Okamoto, Hideaki Ishigami, Kumiko Tokimoto, Megumi Matsuoka, Ryoko Tango

**Affiliations:** 1 Department of Early Childhood Education, Women’s Junior College of NSSU, Tokyo, Japan; 2 Faculty of Sports Science, Nippon Sport Science University, Tokyo, Japan; 3 School of Nursing, University of Shizuoka, Shizuoka, Japan; 4 Renaissance Inc., Tokyo, Japan

**Keywords:** Parenting program, First-time mother, Emotional distress, Maternal confidence, Propensity score

## Abstract

The purpose of this study is to evaluate the effectiveness of a single session intervention designed to reduce emotional distress in first-time mothers. We held a parenting class for first-time mothers who had given birth at a university hospital in Tokyo, Japan. The program of the class consists of lectures on infant care and group discussion, which is a common form of intervention in Japan. The effectiveness of intervention is assessed according to differences in emotional distress experienced by class participants and nonparticipants, and analyzed by the use of a propensity score method to avoid self-selection bias. In order to be more confident about our results, we employ several variations of this method. Results from statistical analysis show that although the effectiveness of the intervention was limited, it was able to alleviate subjects’ loss of self-confidence as mothers. Because this outcome shows a good degree of consistency across methods, it can be considered robust. Moreover, it is roughly consistent with previous studies. Effectiveness can probably be increased by developing a program that improves upon the intervention.

## Introduction

Persistent crying in infancy frequently occurs at 2–3 weeks of age, continuing for up to 3 months and peaking at 1–2 months [1, 2]. Most mothers experience difficulty in coping with their infants when they cry for unknown reasons. That situation causes high levels of emotional distress to mothers, and especially for new mothers, it can lead to loss of self-confidence in their parenting skills [3, 4]. As Percival suggested [5], such distress of first-time mothers can be reduced by supportive intervention from a parenting expert during the period soon after birth. Following this suggestion, we attempted to reduce emotional distress of first-time mothers through intervention in the form of an early parenting class designed to help them better understand infant crying and to provide advice on appropriate behavioral responses. Parenting classes are a form of intervention widely conducted in Japan. For an example of parenting classes in Japan, see Goto et al. [6]. Home visiting programs by health professionals such as public health nurses and midwives are also widely implemented as another form of intervention.

Evaluation of intervention effectiveness required estimation of the differences in emotional distress between the intervention group and the control group. The ideal way to do this would be to conduct randomized controlled trials (RCT) where first-time mothers are randomly assigned as participants or non-participants, but that would present an ethical problem. Instead, we use the propensity score method to avoid self-selection bias. Prior studies of parenting programs implemented in Japan [6, 7] did not use the propensity score method even though RCT was not feasible. Therefore, their results might be biased. To the best of our knowledge, this is the first attempt to use propensity score in evaluation of a parenting program. The objective of this study is to find and evaluate the causal effects of an intervention aimed at alleviating maternal emotional distress.

## Methods

### Subjects

The subjects were first-time mothers who gave birth at a university hospital in Tokyo between July 2009 and February 2010. Some mothers were not accepted as possible subjects, according to the following exclusion criteria: birth was multiple or premature, mother received mental illness diagnosis, or infant required postpartum hospitalization. Upon hospital discharge, all of the mothers who did not meet the exclusion criteria received an explanation of our research verbally and in writing. They were informed that a single-session parenting class would be offered each mother at 1–2 months postpartum, that participation was voluntary, with no penalty for non-participation, and they received an explanation of how the information we collected would be handled. At the request of the ethics committee of the university hospital, we allowed mothers who refused to answer a questionnaire for our study to attend the class. Mothers agreeing to be subjects in the study were selected for the sample. They received an anonymous, self-administered questionnaire at 2 or 3 weeks postpartum by postal mail. A second survey of all subjects, including those who had not participated in the parenting class, was conducted 3 months after the intervention. The questionnaires were numbered to allow for follow-up of the subjects. All procedures in the study were conducted in accordance with the Helsinki Declaration, and were approved by the ethics committee of the university hospital where the study took place.

We recruited 148 first-time mothers as a result of the procedure. A total of 79 mother-infant pairs attended class sessions accommodating 10–15 pairs each, while 69 chose not to attend. Those who refused to answer the questionnaire included 40 participants and 2 nonparticipants. Survey questionnaires were sent to 106 mothers, consisting of 39 participants and 67 nonparticipants. After eliminating 9 individuals with missing data (listwise deletion), 97 mothers remained in the data set. Three of the nine mothers eliminated from the data set were participants.

### Outline of the Parenting Class

The early parenting classes were held during the daytime on weekdays at the university hospital from August 2009 to March 2010. It was not possible to hold the classes on weekends, due to restrictions on the use of facilities. Nowadays in Japan parenting classes are commonly held during the daytime on weekdays, usually at local public health centers or maternity clinics.

The program of the early parenting class covered the following three topics:

1. Changes in crying patterns throughout infant development
2. Approaches to coping with infant crying
3. Regional parenting support resources

Instructors of the class were midwives working at the university hospital. They were given training to conduct the class according to a script in order to avoid differences between class sessions. They explained the topics using a 10-page pamphlet we prepared and demonstrated how to soothe a crying infant. The pamphlet contained many attractive illustrations accompanied by a minimum of descriptive text, to assure that the mothers would be able to read it easily. The program was 3 h long, including breaks and a discussion session. The discussion’s objective was sharing of viewpoints on worries about parenting. The mothers took the pamphlet home for later reference.

Parenting classes in Japan generally offer mothers in the first few months postpartum information on such topics as maternal or infant nutrition, accident prevention and relaxation skills to manage stress. The topics we selected for the class were mainly related to infant crying as one of the factors associated with maternal confidence.

### Survey Items

The questionnaire was designed on the basis of our prior research [8] and advice from maternity nursing specialists and midwives. In addition, a pilot survey was carried out on 10 mothers having infants aged 3 months. The questionnaire consisted of items measuring emotional distress resulting from child care, characteristics of the mother and infant, and effective support received.

#### Outcome Measures

The following four items are designed to assess emotional distress in mothers. The subjects were asked to report on how they experienced distress when their infants cried:

1. “I was afraid the baby would never stop crying” (hereafter abbreviated as “Endless”)
2. “It was irritating.” (“Irritation”)
3. “It shook my confidence as a mother.” (“Confidence”)
4. “I felt helpless.” (“Helpless”)

The magnitudes of maternal emotional distress are measured using the VAS (visual analogue scale). The reliability of VAS is checked by a test and a retest using a pilot survey sample. The correlation coefficients of 0.92–0.97 indicate strong correspondence that confirms the stability of the VAS measurements. In the following analyses, the above four types of emotional distress are treated as outcome variables.

#### Characteristics of Mothers and Infants

We used maternal age, marital status, working status, participation in a prenatal class, the infant’s gender, and the infant’s birth weight as characteristics. Prenatal classes are generally held by hospitals or health centers administered by local governments and usually take the form of a series of lectures by a midwife on the childbirth process and the woman’s nutrition.

#### Parenting Situations

Current parenting situations were assessed by the following four questions. Answers were scored on a 5-point scale from 1 = “No, not at all” to 5 = “Yes, very much.”

1. “Have you experienced persistent crying of your child this week?” (“Crying”)
2. “Have you felt fatigue recently?” (“Fatigue”)
3. “Do you feel anxiety about the financial burden of raising your child?” (“Financial burden”)
4. “Do you think you are knowledgeable about infant crying?” (“Knowledge”).

#### Effective Support

Using a 5-point scale from 1 = “No, not at all” to 5 = “Yes, very much so,” we assessed whether a mother had effective support on the basis of the following three items:

1. “Is there a good source of information on parenting support available to you where you live?” (“Information”)
2. “Do you share parenting responsibilities with someone?” (“Sharing”)
3. “Are you satisfied with help received from the person sharing in parenting?” (“Satisfaction”)

In the following analyses, the above ordinal categorical variables are treated as continuous variables.

### Estimating the Propensity Score

The propensity score analysis proposed by Rosenbaum and Rubin [9, 10] is a statistical technique that estimates causal effect of treatment under conditions in which RCT is difficult to implement for ethical or practical reasons. It has been applied in various research fields (see recent survey papers [11–13] and the textbook by Guo and Fraser [14]). We employ several variations of the propensity score method that are available to be more confident that the results are robust.

In non-experimental studies, the true value of the propensity score is not known and must be estimated using the study data. We followed the common practice of applying logistic regression to estimate a propensity score. To determine which variables to include in a logistic regression, several points must be taken into consideration. First, in an observational study using a propensity score method, the purpose of estimation in the logistic regression model is not to precisely estimate probability of assignment to a particular treatment, but to get a variable used to balance on covariates. Therefore, statistical criteria such as Hosmer–Lemeshow test statistics or AUC (the Area Under the ROC Curve), are not informative [15, 16]. Secondly, all variables expected to show a relationship to the outcome should be included, regardless of whether they are significantly related to the assignment [17]. Taken together, variable selection should be based on prior subject matter knowledge, not on any statistical criteria. Thirdly, as Schafer and Kang [12] suggested, in a study like ours that uses a small sample size, it may be necessary to be frugal in selecting covariates (see Weitzen [18] on sample size in propensity score analysis). Lastly, as shown in Steiner et al. [19], the choice of covariates has a much stronger impact on bias reduction than the choice of a specific method for the estimation of any treatment effect. Thus, we estimate the treatment effects using two sets of covariates, and thereby we check the sensitivity of the estimates to the choice of covariates.

The initial (before intervention) values of the four outcomes are commonly included in both sets because they can be expected to have an effect on outcomes independent of participation in the early parenting class. Other variables used should be associated with the outcomes: participation in prenatal class, working status, “Satisfaction,” and “Crying.” Of four variables, significant differences between the groups are shown for working status. Accordingly, two covariate sets, “Set A” not including, and “Set B” including working status, are established. Our logistic regression model does not include any interaction terms. Although our study has four outcomes for which separate propensity scores could be constructed, we followed precedent [20–22], constructing a single propensity score and applying it to the four outcomes. The next section gives short descriptions of each method used in this study. See references cited therein for details.

### Various Methods for Estimating Treatment Effect

#### Full Matching

Matching is a procedure that creates a new sample of cases to reduce covariate imbalances between the groups. First proposed by Rosenbaum [23] and later developed by Hansen [24], full matching is a way of overcoming the drawbacks of nearest neighbor matching. An accessible description of the method is given in Stuart [21]. We used the R package optmatch (Hansen and Klopfer [25]) to create a matched sample. After creating a matched sample, it is necessary to assess the similarity in covariate distribution. Another point concerning the matching procedure is choice of the width of the caliper. Austin [26] recently recommended setting the width at 0.2 of the standard deviation of the logit of the estimated propensity score. Applying a smaller caliper should reduce the bias, but may also reduce the number of matched subjects and increase variance of estimated treatment effect. If a value of 0.2 was employed, covariate sets A and B would not match for 26 and 25 subjects, respectively. Because that would amount to a substantial reduction of a small sample size like ours, we set the width of the caliper to 0.3. Using this value, the number of unmatched subjects becomes 15 and 24 for sets A and B, respectively. Furthermore, there is an argument for the method of testing treatment effect. Austin [27] argues that matched samples can be assessed using the paired *t* test (or Wilcoxon signed rank test), whereas Schafer and Kang [12] maintain the unpaired *t* test (or Wilcoxon rank sum test) should be used. Accordingly, we performed both tests, and present the results. Even after matching, imbalance of covariates may remain. It has been proposed that it may be possible to eliminate remaining bias by incorporating regression into the matching analysis [12, 28, 29]. Note that the estimated propensity score itself is not included in the linear regression model.

#### Inverse Propensity Weighting

This is a multivariate analysis using propensity scores as sampling weights (see [30, 31] for details). The weighting estimator we employed here is *Δ*_IPW2_, using the notation given in Lunceford and Davidian [31]. As with the matching method, effect of treatment can be estimated by combining weighting with regression to remove any remaining bias. The coefficients in the linear regression model are now estimated by weighted least squares.

#### Doubly Robust Estimation

Doubly robust estimation is a method that specifies two models simultaneously and produces consistent parameter estimates if either of the two has been correctly specified (see [31–33] for details). One model is the relationship between assignment of treatment and covariates. The other is the relationship of outcomes to the covariates and the intervention.

## Results

### Descriptive Statistics

Table 1 presents the descriptive statistics for the subject characteristics, with the last column showing *p* values of the tests for differences in means and proportions between participants and nonparticipants.

**Table 1:** Descriptive statistics

Variable	All (*N* = 97)	Non-participant (*N* = 61)	Participant (*N* = 36)	*p*-Value
*Emotional distress*				
Endless (before)	4.41 (3.10)	4.09 (3.02)	4.96 (3.18)	0.19
Endless (after)	2.64 (2.54)	2.19 (2.15)	3.38 (2.97)	0.04
Irritation (before)	3.42 (3.07)	3.51 (3.01)	3.28 (3.22)	0.72
Irritation (after)	3.01 (2.75)	2.81 (2.36)	3.36 (3.31)	0.39
Confidence (before)	4.68 (3.37)	4.31 (3.27)	5.33 (3.48)	0.16
Confidence (after)	2.83 (2.91)	3.11 (2.94)	2.36 (2.83)	0.22
Helpless (before)	3.68 (3.21)	3.30 (3.06)	4.34 (3.39)	0.13
Helpless (after)	2.67 (2.91)	2.43 (2.70)	3.08 (3.24)	0.31
ΔEndless	−1.77 (3.35)	−1.89 (3.06)	−1.57 (3.83)	0.67
ΔIrritation	−0.41 (2.64)	−0.70 (2.76)	0.08 (2.39)	0.15
ΔConfidence	−1.85 (3.46)	−1.20 (3.18)	−2.97 (3.66)	0.02
ΔHelpless	−1.00 (3.00)	−0.86 (2.74)	−1.26 (3.43)	0.56
*Characteristics*				
Age	32.81 (5.32)	32.31 (5.44)	33.67 (5.08)	0.22
Nuclear family	87 (89.7 %)	53 (86.9 %)	34 (94.4 %)	0.40
Working status (working)	44 (45.4 %)	22 (36.1 %)	22 (61.1 %)	0.03
Prenatal class (participation)	73 (75.3 %)	39 (63.9 %)	34 (94.4 %)	0.00
Child’s gender (female)	46 (47.4 %)	28 (45.9 %)	18 (50.0 %)	0.86
Birth weight (kg)	2.98 (0.35)	3.00 (0.33)	2.93 (0.39)	0.34
*Parenting situations*				
Crying (before)	3.31 (1.42)	3.18 (1.42)	3.53 (1.42)	0.25
Crying (after)	2.64 (1.32)	2.56 (1.26)	2.78 (1.44)	0.45
Fatigue (before)	3.91 (0.99)	3.97 (0.84)	3.81 (1.21)	0.48
Fatigue (after)	3.22 (1.14)	3.26 (1.11)	3.14 (1.20)	0.62
Financial burden	3.23 (1.21)	3.11 (1.18)	3.44 (1.25)	0.21
Knowledge	2.62 (1.14)	2.67 (1.19)	2.53 (1.06)	0.54
*Effective support*				
Information	3.13 (1.28)	3.08 (1.23)	3.22 (1.38)	0.62
Sharing	4.53 (0.84)	4.34 (0.98)	4.83 (0.38)	0.00
Satisfaction (before)	4.13 (1.04)	3.98 (1.15)	4.39 (0.77)	0.04
Satisfaction (after)	4.18 (0.98)	4.10 (1.04)	4.31 (0.86)	0.29

Continuous variables are reported as mean (standard deviation). Dichotomous variables are reported as *n* (%)

Δ indicates the difference between before and after the intervention

“Before”/“after” mean “before intervention”/“after intervention”

For continuous variables and categorical variables with five levels, *t* tests were conducted

For dichotomous variables, χ^2^ tests were conducted

All of the sampled mothers are married, and the mean age is 32.8 years. This is older than the mean age of 29.7 years for first-time mothers in a nationally representative survey taken in 2009 (Ministry of Health, Labour and Welfare, [34]). Since the sample used in this study was taken at a university hospital located in a major urban center, this difference is accounted for by the fact that average age of first-time mothers in urban areas is consistently exceeded by that of their counterparts in rural areas in present-day Japan. Another factor is that the hospital in our study has been actively involved in assisted reproductive technology.

Systematic differences were observed between the groups for several variables, including “Endless” (after intervention), changes in “Confidence,” working status, participation in the prenatal class, “Sharing,” and “Satisfaction” (before intervention). It should be noted that all of the employed mothers in the sample were on parental leave.

### Estimated Propensity Score

**Fig. 1:**
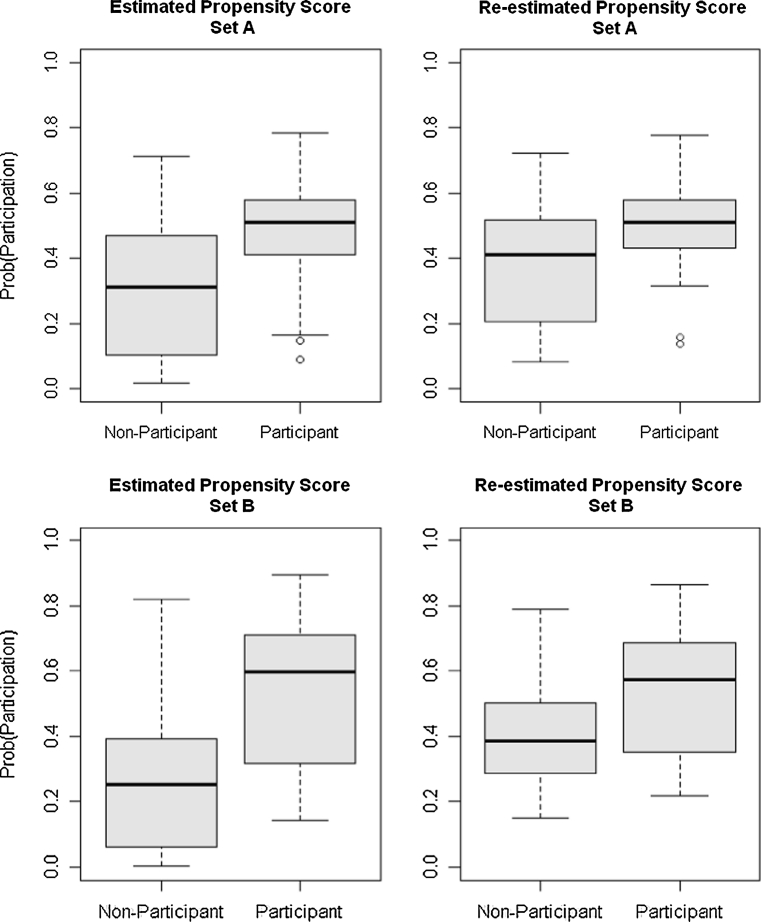
Estimated propensity scores

The estimated propensity scores shown as two boxplots in the left side of Fig. 1 show intervals in which propensity scores do not overlap. This situation, known as the common support problem, can lead to imprecise estimates. Among the various more or less ad hoc proposals for solving this problem, Crump et al. [35] proposed a systematic method involving a rule of thumb by which discarding the subjects with estimated propensity scores outside the range [0.1, 0.9] show a good approximation to an optimal rule. Employing this method reduces the sample size from 97 to 82 for covariate set A and to 76 for set B. Sample sizes of 82 and 76 may be too small for propensity score analysis, so we estimated the effects using all data and took the results for the restricted sample as additional evidence. The boxplots of the re-estimated propensity score for the restricted sample are shown in the right side of Fig. 1.

### Estimation of the Intervention Effect

Table 2a, b present the standardized differences and variance ratios. These measures for checking imbalances on covariates have been used frequently in recent analyses [36]. Balance is achieved when the standardized difference is close to 0 and the variance ratio is close to 1 for each covariate and propensity score. Some researchers, e.g., Austin et al. [37], have suggested that a standardized difference greater than 0.10 represents meaningful imbalance, whereas Harder et al. [38] provide 0.25 as a guideline. To date, no consensus has been formed on an indicator for success of the matching procedure. As seen in the tables, it does appear that balance is achieved in the caliper matched and restricted samples, while imbalances of covariates may remain for the full sample.

**Table 2:** Standardized difference and variance ratio

Covariate	Standardized difference				Variance ratio			
	Original sample	Full sample	Caliper matching	Restricted sample	Original sample	Full sample	Caliper matching	Restricted sample
(a) Covariate set A								
Prenatal class	0.80	0.12	0.19	0.05	4.34	0.93	0.80	0.91
Satisfaction	0.42	0.56	0.29	0.21	2.24	2.42	2.27	1.67
Crying	0.24	0.42	0.18	0.13	1.00	0.78	0.82	1.03
Endless	0.28	0.27	0.16	0.16	0.90	0.99	0.95	1.02
Irritation	0.08	0.30	0.34	0.13	0.87	1.18	1.00	1.00
Confidence	0.30	0.27	0.14	0.13	0.88	0.89	0.86	1.02
Helpless	0.32	0.12	0.12	0.05	0.82	0.95	0.98	1.29
Logit-PS	1.02	0.14	0.00	0.10	3.24	1.37	0.89	1.19
(b) Covariate set B								
Working status	0.51	0.41	0.04	0.07	0.96	1.06	1.01	1.02
Prenatal class	0.80	0.09	0.08	0.09	4.34	0.93	0.77	1.47
Satisfaction	0.42	0.45	0.22	0.00	2.24	2.32	1.33	1.62
Crying	0.24	0.29	0.05	0.09	1.00	0.71	0.96	1.10
Endless	0.28	0.34	0.06	0.04	0.90	0.97	0.74	0.95
Irritation	0.08	0.36	0.50	0.07	0.87	0.98	0.73	0.64
Confidence	0.30	0.42	0.08	0.11	0.88	0.81	0.72	0.85
Helpless (1)	0.32	0.28	0.18	0.09	0.82	0.91	0.82	0.85
Logit-PS	1.26	0.32	0.12	0.05	2.48	2.27	1.07	0.95

“Logit-PS” is the logit of the estimated propensity score

“Full sample” is a matched sample created using full sample

“Caliper matching” is a matched sample created with a caliper width of 0.3

“Restricted sample” is a matched sample based on the reestimated propensity score after discarding the subjects with the estimated propensity scores outside the range [0.1, 0.9]

Table 3a, b show estimates of treatment effects estimated by full matching, and Table 4 shows them by full matching with regression adjustment. From the results, “Confidence” was affected significantly by the intervention. The estimates obtained by the method of inverse propensity weighting are shown in Table 5. The results are the same as that obtained by full matching. Table 6 presents doubly robust estimates, where standard errors are obtained using formula (21) in Lunceford and Davidian [31].

**Table 3:** Estimated treatment effect with full matching

Outcome	Estimate	Unpaired *t* test	Rank sum test	Paired *t* test	Signed rank test
(a) Covariate set A					
*Full sample*					
Endless	0.5432	1.185	0.685	1.127	0.681
Irritation	−0.0616	0.157	−0.281	0.172	0.065
Confidence	−1.7462	−3.008**	−3.030**	−4.055***	−3.309***
Helpless	−0.4075	−0.531	−0.966	−0.565	−0.638
*Caliper matching*					
Endless	0.5816	1.378	0.803	1.358	1.063
Irritation	−0.0416	0.238	−0.219	0.258	−0.009
Confidence	−2.1993	−3.104**	−3.026**	−3.667***	−3.172***
Helpless	−0.6883	−0.306	−0.939	−0.322	−0.911
*Restricted sample*					
Endless	0.7049	1.372	0.969	1.280	1.117
Irritation	0.3102	0.454	0.156	0.423	0.300
Confidence	−2.2603	−2.688**	−2.953**	−3.002**	−2.763**
Helpless	−0.4332	−0.398	−0.865	−0.398	−0.613
(b) Covariate set B					
*Full sample*					
Endless	0.5610	0.940	0.528	1.065	0.548
Irritation	0.0437	0.620	0.319	0.819	0.441
Confidence	−1.7692	−2.842**	−3.267***	−2.634*	−2.677**
Helpless	−0.4466	−0.220	−0.770	−0.237	−0.624
*Caliper matching*					
Endless	0.0337	0.768	0.449	0.777	0.711
Irritation	−0.5726	−0.070	−0.312	−0.067	−0.114
Confidence	−2.3171	−3.192**	−3.252***	−4.244***	−3.391***
Helpless	−0.9881	−0.718	−1.064	−0.785	−1.575
*Restricted sample*					
Endless	0.8405	0.680	0.329	0.732	0.276
Irritation	0.5348	0.711	0.176	0.805	0.536
Confidence	−1.7139	−2.617*	−2.927**	−2.689*	−2.321*
Helpless	−0.1607	−0.268	−0.658	−0.297	−0.471

Values other than estimates are test statistics for each test

***, **, and * indicate significant at 0.1, 1 and 5 % levels, respectively

**Table 4:** Full matching with regression adjustment

Outcome	Covariate set A			Covariate set B		
	Estimate	SE	Robust	Estimate	SE	Robust
*Full sample*						
Endless	0.6355	0.421	0.473	0.9649	0.438	0.493
Irritation	0.2148	0.335	0.339	0.5809	0.376	0.391
Confidence	−1.8782	0.378	0.422***	−1.4508	0.457	0.560*
Helpless	−0.4613	0.415	0.456	0.0713	0.444	0.524
*Caliper matching*						
Endless	0.6063	0.499	0.531	0.0838	0.587	0.588
Irritation	0.2265	0.397	0.409	−0.1817	0.485	0.535
Confidence	−2.2799	0.449	0.467***	−2.2188	0.460	0.463***
Helpless	−0.7723	0.501	0.537	−0.7052	0.529	0.574
*Restricted sample*						
Endless	1.0268	0.510	0.506*	1.1220	0.543	0.546*
Irritation	0.5216	0.528	0.535	0.5415	0.470	0.490
Confidence	−2.0321	0.509	0.517***	−1.6696	0.515	0.562**
Helpless	−0.0815	0.562	0.534	−0.0189	0.533	0.562

Estimates other than parameter that represents the treatment effect are not shown for saving the space

Column labeled “Robust” shows heteroskedasticity-robust standard errors

Hypothesis tests are based on robust standard errors

***, ** and * indicate significant at 0.1, 1, and 5 % levels, respectively

**Table 5:** Inverse propensity weighting

Outcome	Covariate set A					Covariate set B				
	IPW		IPW with regression adjustment			IPW		IPW with regression adjustment		
	Estimate	SE	Estimate	SE	Robust	Estimate	SE	Estimate	SE	Robust
*Full sample*										
Endless	0.7531	0.454	0.7176	0.479	0.518	0.6237	0.477	0.3211	0.492	0.584
Irritation	0.3092	0.451	0.3190	0.461	0.507	0.4462	0.465	0.3117	0.470	0.545
Confidence	−1.5516	0.499**	−1.7387	0.504	0.611**	−1.4876	0.478**	−1.9120	0.509	0.599**
Helpless	−0.1429	0.499	−0.2820	0.506	0.581	−0.1167	0.471	−0.5251	0.511	0.596
*Restricted sample*										
Endless	0.7383	0.516	0.7048	0.547	0.587	0.5238	0.560	0.5125	0.552	0.598
Irritation	0.4195	0.509	0.3810	0.520	0.570	0.5143	0.549	0.5209	0.542	0.578
Confidence	−1.8777	0.559***	−1.9929	0.565	0.622**	−1.6090	0.570**	−1.6802	0.576	0.620**
Helpless	−0.2790	0.570	−0.3758	0.587	0.648	−0.1442	0.565	−0.1946	0.574	0.609

*** and ** indicate significant at 0.1 and 1 % levels, respectively

**Table 6:** Doubly robust estimation

Outcome	Covariate set A		Covariate set B	
	Estimate	SE	Estimate	SE
*Full sample*				
Endless	0.8335	0.472	0.5007	0.492
Irritation	0.2502	0.448	0.1857	0.440
Confidence	−1.7479	0.516***	−1.8632	0.528***
Helpless	−0.3743	0.504	−0.6056	0.523
*Restricted sample*				
Endless	0.7519	0.538	0.4808	0.559
Irritation	0.3344	0.516	0.4795	0.522
Confidence	−2.0328	0.556***	−1.6921	0.600**
Helpless	−0.4277	0.575	−0.2273	0.585

*** and ** indicate significant at 0.1 and 1 % levels, respectively

A total of 24 estimates are obtained for each outcome. From the estimation results, it is clear that the types of emotional distress labeled “Irritation” and “Helpless” were not affected by intervention. Furthermore, “Endless” also could not have been affected because only two of 24 estimates are significant. Although intervention could not alleviate these three types of emotional distress, it can be considered to have reduced the “Confidence” category because all of the estimates are significant and they are all similar to each other. We can conclude that the intervention had an effect on maternal psychological distress, but only for the “Confidence” category.

## Discussion

### Strength of the Intervention Effect

We begin by examining the effectiveness we found for intervention in the “Confidence” category of our four types of emotional distress. We then consider the other three cases in which no effectiveness was determined and how intervention should be redesigned to increase its effectiveness.

As Pauli-Pott et al. [39] reported, mothers frequently interpret persistent infant crying as a negative response by their infants. Though crying does not necessarily signify rejection of care received, mothers can nevertheless feel their maternal confidence shaken when they fail to calm their infants down. It is reasonable to presume this tendency to be particularly strong in first-time mothers. Since first-time mothers have little opportunity to know about the nature of infant crying, they may not fully appreciate how crying fits into normal development. An empirical study of Japanese women by Goto et al. [40, 41] demonstrated an association between lack of maternal confidence and being a first-time mother. Bryaton et al. [42] reported that first-time mothers had low parenting self-efficacy during the early postpartum period. Furthermore, it is known that knowledge of infant development is one of the significant factors accounting for the differences in maternal confidence between mothers [43]. Taking these points into account, we used “Confidence” as a measure for assessing the subjects’ knowledge of infant development and their coping skills. The goal of our intervention program was to improve psychological status of first-time mothers by filling knowledge gaps and acquiring parenting skills. From the results of this study, it appears that our attempt achieved this goal in part, through intervention that helped subjects rebuild their parenting confidence.

The strength of the effect shown in our results is similar to that obtained by Barr et al. [44]. Like ours, that study implemented an intervention and examined its effect on maternal knowledge and behavior. The researchers gave to subjects a pamphlet and a DVD that explained strategies for coping with a crying infant. Although the intervention had the effect of increasing maternal knowledge about infant crying, it affected neither the subjects’ behavioral responses to unsoothable crying nor their levels of frustration. Our results suggest that merely providing mothers with information on crying and behavioral responses is not effective in reducing maternal psychological distress. According to Fisher et al. [45], our intervention corresponds roughly to an “educational approach,” which does not focus on the relevant psychological aspects (see also Rowe and Fisher [46]). Because this lack is a possible cause of insufficient effectiveness, programs whose goal is reducing maternal psychological distress should be improved to deal directly with the psychological distress itself. Including the psychological aspects into the program, however, will require a greater number of sessions, which could lessen the program’s effectiveness. We will discuss this point in more detail later.

In addition to the problem of our intervention program, the location of the parenting class was problematical. It was held at the university hospital where the subjects gave birth, instead of a maternity clinic that would most likely be used by women living relatively nearby. Had the class been located in a neighborhood setting, the subjects who attended it would have had a better opportunity to become acquainted with one another and potentially strengthen the parenting network of mothers in the neighborhood. As already argued by Kitzinger [3], the presence of similar mothers put a mother in a better position to maintain psychological stability. Thoits [47] emphasizes that support is more effective when it comes from similar others, in the sense of those who share similar experiences, than from significant others, such as family members. An effective intervention in reducing emotional distress in first-time mothers coming from similar others is documented by Dennis et al. [48]. Because intervention in the form of a class held at a university or general hospital is relatively inconducive to subjects developing mutual relationships, it is desirable that intervention be done so that the subjects are from the same residential area or that the class be held at a local public health center. One important role now played public health centers is supporting and promoting the formation of community groups of mothers with infants or toddlers. One of the objectives for holding a parenting class is bringing mothers together and helping them bond with one another.

Next, intervention in the present study only concerned mothers. It is hardly necessary to point out that the relationship between the parents is important in parenting. For example, Mulsow et al. [49] examined which predictors of maternal stress were significant for each parenting stage, and found that intimacy with the partner was a significant factor during the early postpartum period, up to about 6 months. Midmer et al. [50] is a successful example of targeting couples for prenatal intervention. Intervention by Fisher et al. [45] targeted not only mothers but also their partners, successfully obtaining their greater understanding and empathy for the mothers. It appears that for an intervention strategy to be effective in reducing emotional distress of mothers, some kind of measures targeting the mothers’ partners should be part of the package.

There are, however, some practical difficulties in addressing this point. First, since fathers’ take-up rate of parental leave is only 2.63 % as of 2011 in Japan [51], it is difficult for them to participate in our class on weekdays. One recent study of Japanese women similar to ours, by Fujiwara et al. [7] did not even include fathers as targets for the parenting program. If we held the class on weekends, we could expect fathers’ participation. The second problem is associated with this point. As noted earlier, the parenting class was held in cooperation with a hospital that, for reasons of its own, was reluctant to hold the class on weekends. Our intervention must be conducted within the limits of these circumstances.

Finally, since our sampled mothers participated only once in the class at postpartum, insufficient effectiveness might be due in part to inadequate instruction time. Thus, intervention consisting of a series of classes is conceivably a way to increase effectiveness, but that is not necessarily true. Reid et al. [52] cast doubt on the notion that “more is better,” and Sanders [53] points out that the time needed to complete the program is a factor that influences a subject’s willingness to participate. More and longer sessions would impose a greater burden on the subjects, so attendance might drop, making the program that much less effective. When we plan a parenting program, we must take into account the burden to mothers participating in several classes 1–2 months postpartum. In an example of a parenting program with a single session, Matthey et al. [54] confirmed that it was effective in reducing postpartum distress in first-time mothers with low self-esteem. In contrast, Matsumoto et al. [55] showed that a program of five sessions, whose subjects were Japanese parents with toddlers or young children living in Australia, had the effect of strengthening their confidence. It was, however, unsuccessful in reducing anxiety or stress. Taken together, it is still unclear whether a program consisting of multiple sessions is more effective than a single session program. Although the effect was limited, our study demonstrated that a parenting program with a single session had a positive effect on maternal confidence. Future studies will design intervention programs taking these points into consideration.

### Limitations

This study has several limitations. First, because the size of the sample is rather small for propensity score analysis, there is some uncertainty as to the assessment of treatment effectiveness. Second, the difference in mean age between the sampled mothers and the national representative survey suggests that the subjects of the present study were not representative of the population of first-time mothers. The fact that the subjects of this study all came from a university hospital in an urban center may account for the difference. In a study conducted in Vietnam, Goto et al. [41] noted that mothers delivering at a university hospital had a relatively high socioeconomic status. The same is probably true in Japan. Future studies estimating the causal effects of intervention will sample subjects from a more representative variety of maternity facilities.

## Conclusions

This paper described the content, implementation and assessment of an intervention aimed at reducing the psychological distress of first-time mothers during the early postpartum period. The intervention consisted of a class for subjects intended to provide them with knowledge about and skills for parenting. Since the mothers’ participation in the study was voluntary, we used the propensity score method to correct for self-selection bias, and showed the potentiality of the method for evaluating a parenting program. Propensity score analysis of the results indicates partial success in reducing the subjects’ psychological distress. Although effectiveness was limited, it is encouraging that the intervention can work. Our analysis suggests that the design of the intervention should be improved in several ways. Implementing improved intervention and statistical evaluation of its effects will be the subject of future studies.
